# Review of CRISPR-Cas Systems in *Listeria* Species: Current Knowledge and Perspectives

**DOI:** 10.1155/2022/9829770

**Published:** 2022-04-23

**Authors:** María del Rosario Espinoza-Mellado, Rodolfo E. Vilchis-Rangel

**Affiliations:** Central de Instrumentación de Microscopia, Depto. Investigación, Instituto Politécnico Nacional-Escuela Nacional de Ciencias Biológicas (IPN-ENCB), Prolongación de Carpio y Plan de Ayala, México City 11340, Mexico

## Abstract

*Listeria* spp. are pathogens widely distributed in the environment and *Listeria monocytogenes* is associated with food-borne illness in humans. Food facilities represent an adverse environment for this bacterium, mainly due to the disinfection and cleaning processes included in good hygiene practices, and its virulence is related to stress responses. One of the recently described stress-response systems is CRISPR-Cas. Clustered regularly interspaced short palindromic repeats (CRISPR) and CRISPR-associated (*cas*) genes have been found in several bacteria. CRISPR-Cas has revolutionized biotechnology since it acts as an adaptive immune system of bacteria, which also helps in the evasion of the host immune response. There are three CRISPR systems described on *Listeria* species. Type II is present in many pathogenic bacteria and characterized by the presence of *cas9* that becomes the main target of some anti-CRISPR proteins, such as AcrIIA1, encoded on *Listeria* phages. The presence of Cas9, either alone or in combination with anti-CRISPR proteins, suggests having a main role on the virulence of bacteria. In this review, we describe the most recent information on CRISPR-Cas systems in *Listeria* spp., particularly in *L. monocytogenes*, and their relationship with the virulence and pathogenicity of those bacteria. Besides, some applications of CRISPR systems and future challenges in the food processing industry, bacterial vaccination, antimicrobial resistance, pathogens biocontrol by phage therapy, and regulation of gene expression have been explored.

## 1. Introduction

*Listeria monocytogenes* is a Gram-positive pathogenic bacterium that can be transmitted through food and it is related with outbreaks [1, 2]. This bacterium has been identified in two different lifestyles: a saprophytic one and a parasitic one, causing a disease known as listeriosis [3, 4]. Listeriosis is considered one of the leading causes of death due to food poisoning, and it is currently estimated that approximately 16% of people that contract listeriosis die each year [5]. The colonization of *L. monocytogenes* in food production environments (FPEs) has been extensively studied, since bacterial control measures are sometimes insufficient due to the pathogenicity mechanisms of *Listeria*, such as the development of a biofilm, which is an ability that is crucial for the survival of this bacterium in food industry environments. [6].

The virulence of *L. monocytogenes* is directly related to invasiveness and its ability to multiply in a wide range of eukaryotic cells [7]. Different virulence factors have been related to these important pathogenicity mechanisms of *L. monocytogenes* [8]. *L. monocytogenes* is classified phylogenetically and genotypically into at least four evolutionary lineages (lineages I–IV). It has been concluded that all lineages have different genetic, phenotypic, and ecological characteristics, which can affect their ability to be transmitted through food and cause human diseases, as well as their ability to thrive in the environment surrounded by phages [9]. *Listeria* has a virulence gene list that extends to noncoding RNA (ncRNA) molecules. One of these ncRNAs is the CRISPR arrays [4].

### 1.1. CRISPR-Cas Basic Concepts

One of the recently described stress-response systems is CRISPR-Cas. Clustered regularly interspaced short palindromic repeats (CRISPR) and CRISPR-associated (*cas*) genes have been found in several bacteria. CRISPR-Cas has revolutionized biotechnology since it acts as an adaptive immune system of bacteria protecting from infection caused by bacteriophages and other genetic elements like DNA, which also helps in the evasion of the host immune response [10–12].

CRISPR-Cas adaptive immune systems are found in roughly 50% of bacteria and 90% of archaea [13–15]. The common structure of a CRISPR locus is a set of *cas* genes and a CRISPR arrangement [16]. Due to complex machinery, small sequences derived from phage DNA (known as spacers) are integrated into a locus of repeat sequences (CRISPR) [17, 18]. The abovementioned statement generates a genetic record of past viral infections [19].

The basic steps in the operation of CRISPR systems are adaptation, expression, and interference [20, 21]. The CRISPR complex demands a protospacer adjacent sequence motif (PAM) to evade the marking of the CRISPR array in order to avoid autoimmunity and self-cleavage. This motif is in the marked DNA [16, 18].

### 1.2. CRISPR Classification

Within the organization of CRISPR-Cas systems, 2 groups have been classified taking into account the Cas complex [11]. Systems that comprise class 1 (types I, III, and IV) employ a set of multi-units and subunits of Cas proteins for the identification of the tagged material, unlike class 2 systems (types II, V, and VI), which use a set of proteins with a single effector for the detection and cleavage. These 2 groups are divided into 6 types and 25 subtypes, which have a great variety of genes *cas* and operon arrangement [14, 16, 22–25]. The type 2 system is known to have a simple CRISPR-Cas organization since it has a distinctive operon scheme, the *cas9* gene and a short trans-activating crRNA (tracrRNA). Therefore, for the interference step, this system only needs *cas9*, tracrRNA, and host RNAse III; the other *cas* genes are not required [17, 18].

## 2. CRISPR Systems in *Listeria*

Different bioinformatic studies show that CRISPR-Cas systems can be found in 10% of different bacterial genomes and approximately 40–50% of bacteria that can be cultured [18, 26], but this has not been demonstrated in experiments *in vivo*. In the case of *L. monocytogenes*, it has been found that 41.4% of some strains contain putative *cas* genes, and in one study, the weak activity of a CRISPR-Cas type 2 system against plasmids containing related spacers and PAM sequences was identified. Nevertheless, the action of this type of systems on *Listeria* against phages has not yet been thoroughly studied since *L. monocytogenes* regularly contains prophage-encoded anti-CRISPR proteins [9, 27]. Moreover, in the case of the species *L. ivanovii*, its operation has not yet been analyzed in detail, but the CRISPR-Cas arrangement has been found, and maybe they play a putative role in the inactivation of invading phage DNA [4, 18, 28].

Three CRISPR loci have been described in the genome of *L. monocytogenes*. Locus 1: described as CRISPR RliB; locus 2 located approximately 10 kb downstream of locus 1, associated with the *cas* subset “*Tneap*” (*cas2*, *cas1*, *cas4*, *cas3*, *cas5t*, *cst2*, *cst1*, *cas6*), which belongs to the CRISPR-Cas type-1B system; and locus 3 located in strain 1/2a EGD-e associated with *cas* subset “*Nmeni*” (*csn2*, *cas2*, *cas1*, *cas9*), which belongs to type-IIA [25, 29, 30]. Di [9] showed that not all strains contained the three CRISPR loci at the same time, so a classification based on CRISPR can be useful to subtype strains of serotype 1/2a (lineage II) and 1/2b (lineage I), but it is limited for many strains of lineage I that show absence of a typical CRISPR structure.

In the study of CRISPR-Cas systems in *Listeria*, a small CRISPR RNA (RliB) has been described in *L. monocytogenes* strain EGD-e. No *cas* genes were found either close to RliB or anywhere else in the genome of that strain. However, despite the absence of Cas proteins, RliB is expressed and significantly increased in bacteria isolated from mice and in bacteria that grow in human blood or that are exposed to hypoxia. Therefore, the authors demonstrated that RliB is involved in the virulence of *L. monocytogenes* and that it binds and is a substrate for polynucleotide phosphorylase (PNPase), which is likely responsible for its processing into a mature form. In bioinformatic analysis, RliB-CRISPR has been discovered in *L. monocytogenes* strains and in other *Listeria* species at the same genomic locus [4, 31].

### 2.1. Anti-CRISPR Proteins in Listeria

The fight for survival between phages and bacteria has resulted in the evolution of different bacterial defense systems and their opponents [32, 33]. Different mechanisms against phages have been reported in *Listeria*, principally in *L. monocytogenes* [34], and anti-CRISPR (Acr) proteins codified by prophages have been described [18, 35]. The role of the Acr proteins is particularly important since it can be directly related to the absence or deficiency of CRISPR-Cas systems in some bacteria due to the integration of prophages in the genetic material of the host and the Acr expression. The first examples of Acr proteins were found in *Pseudomonas aeruginosa*. The anti-CRISPR genes are very important because they represent a mechanism of phages to overcome and inhibit targeting of the CRISPR/Cas systems [13, 16, 36].

So far, 45 groups of Acr proteins have been identified, which are classified into 2 classes. In this last group, the Acr proteins described in *L. monocytogenes* are found. Several *acr* genes have been described to coexist with a conglomerate of genes named “anti-CRISPR-associated genes” (*aca*) [16]. Seven *aca* genes have been identified, and although their exact function has not yet been established, these genes frequently codify a protein with a helix-turn-helix (HTH) motif, which is related to a regulatory function and an N-terminal domain (NTD), whose role has not been described in all the Acr proteins [35]. The *aca* genes have been used to find new Acr proteins and vice-versa [37].

In *Listeria* phages, some anti-CRISPRs proteins, such as AcrIIA1, AcrIIA12, AcrIIA2, AcrIIA3, and AcrIIA4 have been described [23]. AcrIIA1 is a transcriptional autorepressor of the *Acr* locus that is needed for optimal lytic growth and prophage induction [35]. AcrIIA2 and AcrIIA4 could block CRISPR-Cas target DNA producing an effect in the different protein subunits, employing steric or non-steric forms of inhibition, and interfering with guide RNA [16, 32]. These proteins were found to inactivate the type II-A CRISPR–Cas9 proteins of *L. monocytogenes* Cas9 (LmoCas9) and *Streptococcus pyogenes* Cas9 (SpyCas9) *in vivo*. Therefore, these represent potential tools in the toolkit of Cas9-mediated genome editing [38].

**Figure 1:**
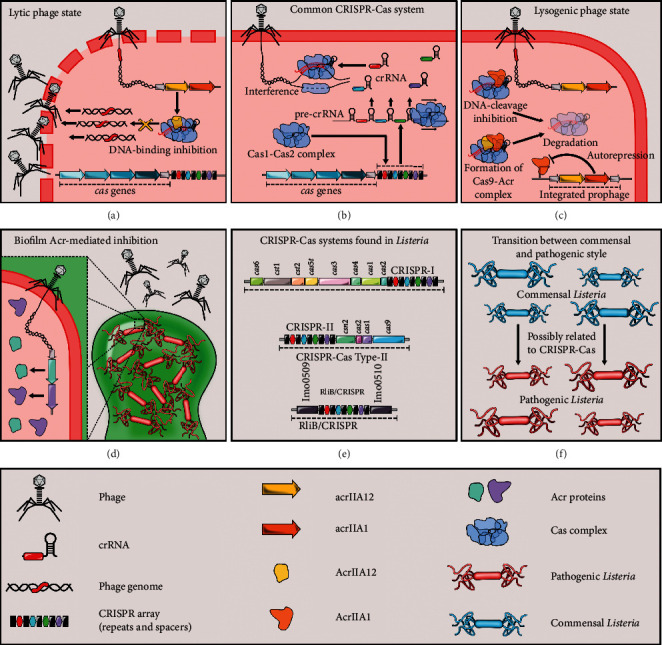
Summary of CRISPR-Cas system and potential applications in *Listeria*. Influence of AcrIIA1 and AcrIIA12 proteins on (a) lytic and (c) lysogenic cycle of *Listeria* bacteriophages. (b) Overview of common CRISPR-Cas mechanism of action. (d) Possible application of Acr proteins mediating biofilm formation and control. (e) Most common CRISPR-Cas systems in *Listeria.* (f) CRISPR-Cas as a potential virulence factor in pathobionts.

Some *Listeria* prophages have developed anti-CRISPR proteins to avoid being degraded. Different *Listeria* phages have been described to inactivate the activity of the CRISPR-Cas system by encoding proteins such as AcrIIA1. This protein can bind to the Cas9 HNH domain to stimulate its degradation and maintain the lysogenic state (AcrIIA1 only binds effectively to Cas9 during the lysogenic cycle, not in the lytic cycle). Phages use the independent protein Acr for their lytic replication (Figure 1) [19]. The study of these proteins is of utmost importance since AcrIIA1 NTD homologs have been identified in some host bacteria, which could lead to an “anti-anti-CRISPR” activity, repressing the display of anti-CRISPR phages, giving a potential advantage to those bacteria [35].

### 2.2. CRISPR in Listeria and Their Relationship with Virulence and Pathogenicity

The role of CRISPR systems goes beyond defense against viruses and plasmid conjugation. One of the applications of the CRISPR-Cas system related to bacterial virulence is to be able to distinguish between species resistant to antibiotics with a wide variety of plasmids carrying resistance genes, from less resistant species since CRISPR interferes with the uptake of phages that carry some virulence genes in many bacteria (e.g., toxins and antibiotic resistance genes) [39]. For example, in the case of *Enterococcus faecium*, the relationship between some clinical strains and the loss of the *cas*1 gene was studied, although all isolates had a type II-A *cas* operon. It was concluded that the increase in antibiotic resistance, as well as phage uptake and pathogenicity islands of this bacterium were due to deletions of the *cas* gene [40]. Another example is enterohemorrhagic *Escherichia coli* (EHEC), in which CRISPR polymorphisms were found to correlate with the presence of two EHEC virulence genes, *stx* and *eae*, which encode the Shiga toxin released by the phage and the intimin virulence factor, respectively. Strains that have lost the CRISPR locus have also been reported to be less invasive or virulent since polymorphisms in genes of associated virulence factors can also be identified with CRISPR [25, 41, 42]. On the contrary, in the case of *Campylobacter jejuni*, it has been observed that the expression of Cas9 in strains that have lost the CRISPR locus increases virulence and that when *cas9* is lost, swarming increases and cytotoxicity is reduced in human cells [31]. Therefore, the abovementioned examples envision a wide field of study to determine whether these characteristics apply to different microorganisms and the full role that CRISPR has with bacterial virulence.

The role of the biofilm as a pathogen and persistent mechanism for *L. monocytogenes* has been described [6, 43–48]. The ability to proliferate in cold and humid environments, as well as the ease of adherence to surfaces, makes *L. monocytogenes* capable of forming biofilms on materials such as plastics, metals, and food [49]. Moreover, the CRISPR-Cas systems could be involved in the regulation of virulence gene expression [25, 26], and it would be interesting to elucidate the relationship between the presence of CRISPR in *Listeria* and the biofilm formation capacity of this bacterium in different materials (for example: prosthetic materials and FPEs) [50–53].

The role of CRISPR during intracellular growth of different bacteria has been demonstrated [25, 54]. This possible relationship of CRISPR with stress-response factors makes the study of intracellular microorganisms such as *L. monocytogenes* interesting [3]. In the case of *Neisseria meningitidis*, Cas9 has been shown to promote its invasion and replication in human cell lines [31]. Furthermore, CRISPR-Cas systems have shown to improve the integrity of the bacterial envelope, promote antimicrobial resistance, and evade multiple innate defense pathways during infection [55].

It has been debated whether CRISPR has importance within commensal microorganisms since it poses a probable regulation of their immune recognition. This could be particularly important in the case of pathobionts, where there is a transition between commensal and pathogenic lifestyle [25, 56, 57]. It has been described that approximately between 0.5% and 5% of the human population carries transiently and asymptomatically low levels of *L. monocytogenes* in the gastrointestinal tract [58–60], and CRISPR could play a role in some stages of this transition process, such as colonization.

## 3. Applications of CRISPR Systems in *Listeria* and Other Bacteria

CRISPR and CRISPR-Cas systems are transforming disciplines such as biotechnology and biology, and various technologies derived from them have been applied in bacteria, although they have been mostly applied in eukaryotes. Some areas in which these technologies have been used are genome editing and gene expression regulation [12, 15, 33]. CRISPR-Cas systems have been used as diagnostic tools, epidemiological, and bacterial evolution studies. CRISPR-Cas systems have been used as a complementary typing tool, for example, in *Mycobacterium tuberculosis*, as a routine genotyping and epidemiology method, or with *C. jejuni* for the study of phylogenetic relationships between strains [11, 33].

CRISPR-based subtyping has been useful for different pathogens, but in the case of *L. monocytogenes*, Taylor and Stasiewicz [61] suggested that the use of CRISPR spacers as a marker to differentiate persistent from sporadic strains are not useful to improve that identification, in comparison with the existing methods, such as Whole Genome Sequencing (WGS) and Single Nucleotide Polymorphism (SNP) subtyping.

The practical application of CRISPR has a wide range of industrial significance and breadth of bacteria where it can be used, such as typing and tracking of bacterial strains (to determine the chronology in the diversification of strains, and therefore, their evolutionary trajectory), bacterial vaccination (important in order to prevent contamination with phages in dairy products), development of antimicrobial agents, and to know the virome that surrounds a bacterial population. One of the future applications of CRISPR is the advanced imaging of bacterial chromosomes due to the specificity and simplicity of Cas9 to tag a specific genomic locus for high resolution imaging. For example, in *L. monocytogenes* has been devised the use of artificial CRISPR arrays to kill pathogenic bacteria by targeting antibiotic resistance or virulence genes [33, 62]. All these techniques can directly impact areas such as the food industry, health, and specific ones, such as bioremediation, biogeography, or biorefineries [17, 20, 25, 26].

Hupfeld [18] demonstrated that by transferring phage constructs based on the CRISPR locus of *L. ivanovii* to other species of the genus, specific bacterial control could be achieved in different co-cultures. Applying it to other species could control and modify complex bacterial environments, by balancing and shaping specific microbiomes as in the stomach. The abovementioned method shows a broad overview of the applications of CRISPR-Cas in this area. Treatment with phages could also be related to one strategy that has been proposed for the control of biofilm formed by *L. monocytogenes* in FPEs in the food industry [6] since CRISPR technology could innovate the control process of this bacterial pathogenicity mechanism.

CRISPR spacers are considered a historical fingerprint of intruders in the host DNA; in the case of *L. monocytogenes*, it has been suggested that strains of a certain lineage could diverge to different genotypes when encountering various invaders, which would be related to evolution between strains of different lineages, depending on specificity of the phage. *L. monocytogenes* has a great importance in the food industry, so the information that the CRISPR system can provide would be an alternative to eradicate or regulate it, due to the function that it has within the bacteria [9].

For most CRISPR-Cas systems from different bacteria, known inhibitors have not yet been described, suggesting that many families of anti-CRISPR proteins have not yet been discovered, so this field of study is quite broad in the future [23]. The use of Acr proteins has been proposed as regulators of the CRISPR-Cas system through an on-off switch process since some of them have been described to inhibit Cas9 activity in cell cultures. Therefore, its use could be optimized for gene editing processes in applications as varied in areas as therapeutics and biotechnology. For example, Acr proteins could benefit gene drive technology, to prevent vector-borne diseases and eliminate pests. Also, some phages already described could exert an inhibition mediated by Acr proteins against multidrug resistant bacteria, resulting in an improvement in the treatment of different diseases [16]. Another application could be the addition of genes encoding Acr proteins to different bacteriophages to be used in biocontrol processes [2], which in the case of *L. monocytogenes* would be very useful in the food industry (Figure 1).

## 4. Conclusions

All the previously described applications of the use of CRISPR within bacterial virulence and pathogenicity in areas as diverse as industry and microbiology give an idea of the powerful strategy that this system must be used in the study of different bacteria. In the case of *L. monocytogenes*, several Acr proteins have been identified, which gives the possibility of using them as a tool for genome editing and identification of “anti-anti-CRISPR” activity It would also be an advantage for these bacteria, which could be linked to increased virulence; also, the study of these proteins could improve the treatment with phages within the food industry or in the environment where *L. monocytogenes* persists and must be eliminated (biocontrol) since the CRISPR system has already been suggested in this bacterium to be effectively labeled and controlled for its determinants of antimicrobial resistance.

More than 30 years have passed since the first description of the CRISPR system, so tools as varied as, for example, the use of Acr proteins, envisage a very promising future to be applied in biocontrol, which is why subsequent studies of the search for CRISPR in isolated strains of different sources will help to recognize all the functions that this system can provide and the potential that it has in order to understand important aspects, such as the evolution of the genus.
